# Design and validation of theory-based perceptions concerning the physical literacy questionnaire for pregnant women (P2LQ-PW)

**DOI:** 10.1186/s12889-022-14204-7

**Published:** 2022-10-23

**Authors:** Leila Kianfard, Shamsaddin Niknami, Farkhondeh Amin Shokravi, Sakineh Rakhshanderou

**Affiliations:** 1 Department of Health Education and Health Promotion, Faculty of Medical Sciences, Tarbiat Modares University, Jalal-e Al Ahmad Highway, Nasr Bridge, Tehran, Iran; 2 Department of Health Education and Health Promotion, Faculty of Medical Sciences, Shahid Beheshti University of Medical Sciences, Tehran, Iran

**Keywords:** Physical literacy, Psychosocial determinants, Pregnant women, Questionnaires

## Abstract

**Background:**

There is evidence that physical literacy plays an essential role in understanding the importance of maintaining appropriate physical activity and thereby preventing illnesses during pregnancy. The present study aimed to design and validate a physical literacy questionnaire focused on pregnant women.

**Methods:**

In this exploratory psychometric study, theory-based perceptions concerning the physical literacy questionnaire for pregnant women were designed. Face-to-face interviews were conducted to develop the initial items. The findings from the interviews were merged with the literature review. The content, construct, and face validity was assessed by the participation of midwives, health educationists, and pregnant women. The content validity ratio, content validity index, and impact score of the items were calculated. The construct validity of the questionnaire was calculated through confirmatory and exploratory factor analysis. The reliability of the questionnaire was calculated by the standard error of measurement, intra-class correlation coefficient, composite reliability, and Cronbach’s alpha.

**Results:**

The highest score was seen in the knowledge subscale and the value of Cronbach’s alpha for the subscales was 0.89 to 0.97, and the value of ICC was 0.76 to 0.89. The correlation according to the opinion of experts was satisfactory in all items of the questionnaire. The minimum loading factor for varimax rotation in the knowledge subscale was 0.41, the attitude was 0.56, the nurture factor was 0.38, and enabling factor was 0.27. The compatibility of the model among the constructs was confirmed by the normed chi-square (χ^2^/ df) < 5.0, comparative fit index ≥0.90, Tucker-Lewis index ≥0.9, and root mean square error of approximation < 0.08.

**Conclusion:**

The physical literacy questionnaire for pregnant women is the first tool based on the PEN-3 model that other researchers can use to collect data and conduct educational interventions to change physical literacy behavior among Persian women during pregnancy.

**Supplementary Information:**

The online version contains supplementary material available at 10.1186/s12889-022-14204-7.

## Background

Pregnancy is an astonishing period of life for a pregnant woman and her unborn infant, so maternal health is a health priority [1]. There is evidence that physical activity plays an essential role in preventing preeclampsia, type 2 diabetes, obesity, and many cancers [2]. Physical activity during pregnancy has many advantages, e.g., a risk reduction of gestational diabetes mellitus (GDM), additional gestational weight gain, and HELP syndrome (hemolysis, elevated liver enzymes, and low platelet), and [3]. This could be individually significant for pregnant women from low and middle-income countries (LMICs), where low socioeconomic status (SES) in circumference has been presented as a risk factor for numerous outbreaks of lack of physical activity, overweight, and impaired glucose intolerance [4, 5].

In the United States, in lack of obstetric complications, as per the guidelines of adults, pregnant women must accumulate 30 minutes or more of moderate-severe physical activity on most, all days a week [6, 7]. The American College of Obstetrics and Gynecology (ACOG) suggested 30 minutes of physical activity 5 days a week for pregnant women [8]. Along with dieting, having physical activity during pregnancy has an effective and impressive intervention for the fetus and maternal health [9, 10]; however, some Iranian pregnant women seem not to follow these guidelines, and a study in Iran showed that 70% of women did not have physical activity during their pregnancy period [11, 12].

It is necessary to mention that having physical activity is one of the essential factors affecting the quality of life. Nowadays, it has increasingly specified that assessing the quality of life can predict the health situation in various groups [13]. Physical activity in the first 20 weeks of pregnancy decreases the dangers of preeclampsia by up to 34% [14, 15]. One of the approaches to increasing physical literacy is using theoretical research to recognize the influences of physical activity on pregnancy. The low range of physical activity during pregnancy may be due to the lack of recognition of theory-based determinants of health promotion [16]. The PEN-3 cultural model is a theory that is used to study the physical activity determinants during pregnancy [17–19]. This model created an opportunity to identify determinant factors that prevent physical activity during pregnancy [17]. This model was also considered to explore how cultural and social systems create a critical role in health subsequence [18]. The PEN-3 cultural model is a framework that concentrates on educational and cultural interventions in health promotion behaviors [19]. This model was used as an instrument to investigate within context and data saturate, analyze, describe, and determine an issue [16]. In addition, the PEN-3 cultural model helps researchers to identify factors that affect behavior [17]. This criterion was designed according to the PEN-3 cultural model framework. In the PEN-3 cultural model, every determinant factor could be measured directly, e.g., by investigating participants about all over their tendency, or indirectly, e.g., by investigating participants about particular behavioral perceptions, nurtures, and enabling factors about physical activity during pregnancy. With this approach, it was common for existing studies to cross-tabulate the Relationships and Expectations (i.e., perceptions, enablers, nurturers) domain with the Cultural Empowerment (i.e., positive, existential, and negative) domain of the PEN-3 model to generate 3 × 3 tables containing nine categories. Commonly for designing and validating a questionnaire based on the PEN-3 cultural model, it has been proposed that for direct scales, one could use the similar direct scales designed by Airhihenbuwa [19].

Some theoretical questionnaires measure the determinant factors of physical activity among pregnant women presented in the literature [20]. Nevertheless, there are no general validated scales to measure perceptions, nurture and enable pregnant women to exercise during pregnancy. Thus, the designed tool could help researchers to plan interventions for increasing physical literacy in pregnant women. It has been shown that people with physical literacy do more physically active than those without [21, 22]. In the United States, 25% of people do not have physical activity in their workplace, and 12% of total mortality was attributed to low mobility [23]; however, this study was limited to women with no children (nulliparous women). The Iran studies show that males’ inactivity lifestyle is less than females [24, 25]. In one study, researchers realized that abdominal obesity was approximately six times more common in females than in males [26].

Although physical activity may not be prominent in the first trimester of pregnancy, as the pregnant woman’s body varies quickly, it may be valuable to recognize the cultural model determinants of physical literacy during pregnancy. This study evaluates psychosocial and perceptual (knowledge, attitude) determinants based on the PEN-3 cultural model about the reason for lack of physical activity in pregnant women for utilization in the second and third periods of pregnancy. This study aimed to design and validate theory-based perceptions concerning the physical literacy questionnaire for pregnant women (P2LQ-PW).

## Method

### Research design, context, and participants

The present exploratory psychometric study was conducted in Tehran City, Iran, from January to March 2019. The sample size was estimated at 440 persons (Confidence = 0.95; Power = 0.9), and to compensate for loss, 480 nulliparous pregnant women in two or three trimesters of pregnancy that were between 28 and 35 years, had no background of severe complications of the disease, and without any physical activity during pregnancy were selected randomly from selected health centers of the 5th district of Tehran City, Iran [27]. Of the 480 participants, 30 dropped out of the study.

Table 1 summarizes the details related to the participants in each phase, which indicates the recruitment of different participants.

**Table 1:** Details for the participants in each phase of the study

Phase of study		Participants’ job and number male(m) + female(f)				Assessment measure or target
		Pregnant Women	Health educationist	Midwives	Experts from a health promotion institute	
**Item generation**	Interviews	6f	4(1 m + 3f)	4(f)	4(1 m + 3f)	Identifying the factors influencing preventive behavior of pregnant women
	Group discussion	0	3f	3f	3f	Finalizing the first draft of the research questionnaire
**Assessment of face validity**	Qualitative way	15f	7(2 m + 5f)			Resolving ambiguity in meaning, wording, grammatical errors, and allocation of the items
	Quantitative way	15f	0	0	0	Calculating IS of the items
**Assessment of content validity**	Qualitative way	0	7(2 m + 5f)	7f	3f	Calculating CVR of the items
	Quantitative way	0	7(2 m + 5f)	7f	3f	Calculating CVI of the items
**Assessment of construct validity**	EVA	225 f	0	0	0	Calculating KMO of the questionnaire; factor loadings
	CFA	225 f	0	0	0	Calculating fit indices of the structural pen-3 model
**Reliability assessment**		42 f	0	0	0	Calculating Cronbach’s α, ICC, SEM, CR of the questionnaire

In this study, selecting specialists with valuable articles or having high experience in this field is considered necessary. Therefore, the experts were invited from different groups to help generate items, categorize and finalize the questionnaire’s first draft, and the items’ content validity [28, 29]. Additionally, all the pregnant women were female, and the details of their selection for this study in each phase are described as follows.

### Developing the first draft of the questionnaire

The first items of P2LQ-PW were achieved through literature review and interviews with all pregnant women. Furthermore, databases including PubMed, EMBASE, MEDLINE, Cochrane Library, and Allied Health Literature (CINAHL) were searched to find the studies published about physical literacy in pregnant women. In this regard, a combination of the keywords of “Physical literacy, “Psychosocial Determinants,“ and “ Pregnant Women was utilized to explore in English and Persian. Thus, 714 articles issued between 2006 and 2020 were obtained, and their abstracts were read. Then, ten Persian and five English questionnaires were obtained by reviewing the articles and contacting the corresponding authors [30, 31]. This survey was necessary to extract the cultural background of different societies and need assessment for developing and designing the present questionnaire.

All the interviews were investigated to identify the influencing factors of the physical activity behavior in pregnant women. The pregnant women, health educationists, midwives, and experts participated in 30-45 min face-to-face interviews in their desired time and place. They were told that their information would be kept confidential and used anonymously. At the end of the interviews, the items obtained from the literature review results were combined with the interviews. Furthermore, health educationists, pregnant women, and experts were asked to participate in two focus group discussions. Each session lasted 90 minutes, one research team member acted as the session’s coordinator, and another took note. In addition, a directed analysis was performed on the content of the interviews, some questions were deleted, and some of the questions were edited. Finally, the research team members confirmed and finalized the first draft of the study P2LQ-PW.

### Assessment of face and content validity of the questionnaire

The face validity of the intended P2LQ-PW was examined qualitatively and quantitatively by cooperating with 50 pregnant women, 10 health educationists, 8 panelists, and 10 midwives. In qualitative evaluation, any ambiguity in the meaning, wording, and scaling of the items and grammatical errors and those in item allocation were identified and resolved based on feedback from pregnant women and health educationists. However, each item’s impact score (IS) was calculated for quantitative assessment. To assess the face validity of the questionnaire, the appropriateness of each item was rated by an expert using a five-point Likert scale. The IS of each item was calculated using the formula of IS = frequency (%) × importance [21, 22]*.* The frequency shows the number of patients rating the item’s appropriateness on a 4 or 5 scale in the study.

The relevance of the questions was also evaluated using a four-point scale: “not relevant,” “insignificant relevancy,” “relevant,” and “reliable relevancy. “Every item’s Content Validity Ratio (CVI) is the ratio of specialists who measure it from 3 or 4 [32]. In this regard, Polite and Beck presented 0.8 for the allowable lower range of CVI value [33]. An appropriate level of the accordance was determined (CVI = 0.91) among panelists proposing that the scale measure had a well content validity.

The necessity of the questions was evaluated using a three-point scale; “not necessary”, “effective but not necessary”, and “necessary”. Then, a Content Validity Ratio (CVR) was calculated for all scales. The necessity of the item in the questionnaire was determined according to the Lawshe table and the number of experts. If more than half of the panelists represent that each item is necessary, that item has a minimum content validity [34].

Therefore, P2LQ-PW was emailed to 20 midwives and health educationists to evaluate the validity. Two experts failed to complete the P2LQ-PW, and one questionnaire was set aside by considering the accuracy of the data. (Response rate = 0.85%).

The CVR was determined based on the three and four-point Likert scales. The formula of (Ne – N/2)/ (N/2) was used to calculate CVR [22], in which N indicates the total number of panelists and Ne illustrates the number of those rating the item as “essential.” However, items with CVR below 0.46 were removed based on the Lawshe table [23].

To compute the CVI of the items, the relevance of each item was rated on a four-point Likert scale using the formula of CVI (the number of specialists who assigned scores 3 and 4 to the items/N). However, the items with a CVI of less than 0.79 were eliminated [24, 25].

### Assessment of the construct validity of the questionnaire

**Fig. 1:**
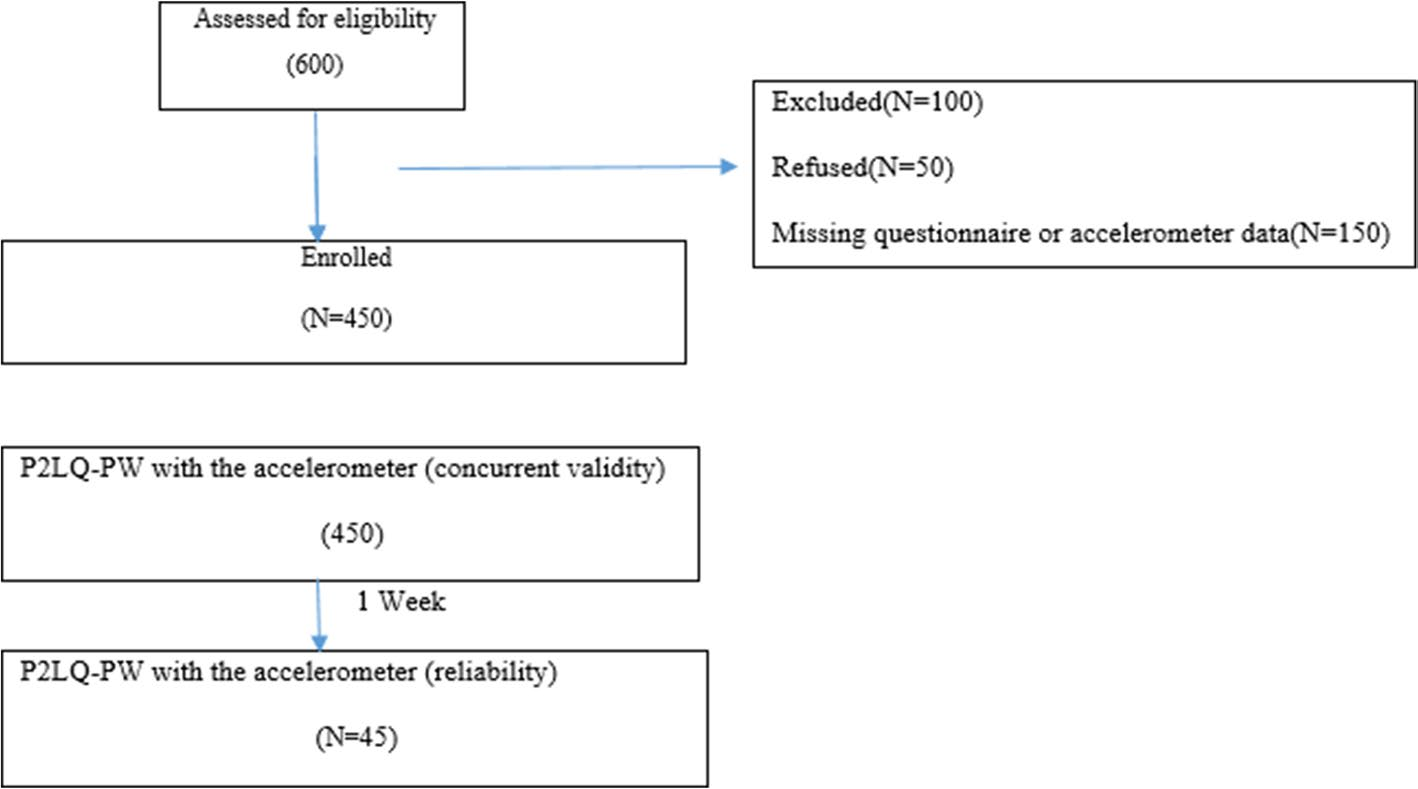
Participant flow diagram for a validation study of the Physical Literacy Questionnaire for Pregnant Women (P2LQ-PW)

The construct validity of P2LQ-PW was examined through exploratory factor analysis (EFA) and confirmatory factor analysis (CFA). Performing each CFA or EFA is recommended by participating at most minuscule 150 from the target group [35]. The sampling framework in this study included 400 pregnant women in Tehran. Due to a 10–15% drop rate in the previous relevant studies, there was a need for 50 more participants [28, 35]. In this stage, 450 pregnant women were randomly selected from Tehran and invited to participate using (www.randomizer.orgsoftware) Fig. 1.

### Exploratory factor analysis (EFA)

The different dimensions of the criterion were specified by implementing exploratory factor analysis (EFA) to apply the oblique rotation and original fundamental factoring [32]. This study utilized IBM SPSS statistics version 24 for exploratory factor analysis (EFA). Varimax rotation with Kaiser Normalization was chosen as the correlation between factors was less than 0.3 [33]. Because of assessment, sampling efficiency to carry out the satisfactory factor analysis, Kaiser-Meyer Olkin (KMO), Measure of Sampling Adequacy (MSA) for factor analysis, and Bartlett’s test was accounted for. The specific value more significant than one and factor loading equivalent to or larger than 0.4 were used [34].

The validity of convergent was determined to implement correlations of item-scale that reformed for overlaps. It was anticipated that the item scores would correlate greater with a specific assumed scale than other scales. The values of correlation of 0.40 or higher were investigated satisfying (*r* ≥ 0.81–1 as supreme and excellent, 0.61–0.80 excellent, 0.41–0.60 good, 0.21–0.40 fair, and 0.00–0.20 poor) [36]. If an item was loaded into different factors, it was related to the factor that the item had the largest factor loading. After completing the analysis, the items were categorized, and every category formed a factor. The extracted factors were named by members based on the nature of their items and the characteristics they proposed to measure.

### Confirmatory factor analysis (CFA)

**Fig. 2:**
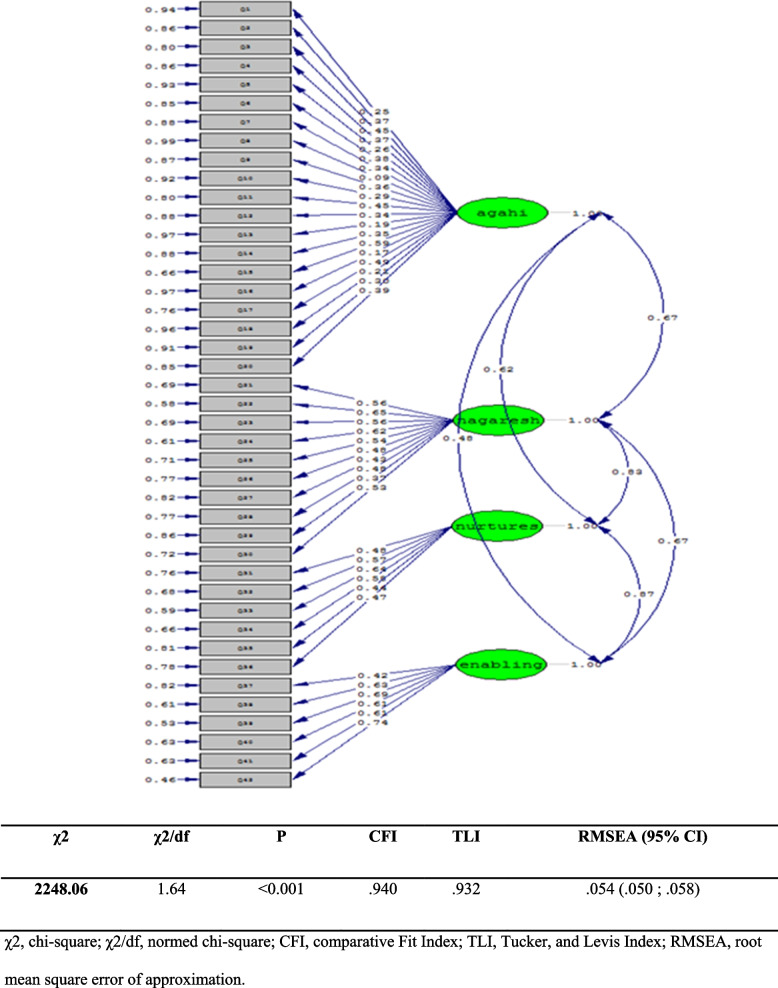
Routing diagrams for generalized validation model for all structures. χ2, chi-square; χ2/df, normed chi-square; CFI, comparative Fit Index; TLI, Tucker, and Levis Index; RMSEA, root mean square error of approximation

The confirmatory factor analysis results for the four sections of the P2LQ-PW showed a χ^2^ value at 3921.78; degrees of freedom, which gave a ratio of χ^2^/dF = 3.51 (*p* < 0.0001). LISREL 8.8 software was used for confirmatory factor analysis (CFA) in the present study. The Comparative Fit Index (CFI), Normed Fit Index)NFI(, and Goodness of fit index (GFI) Values were 0.90, 0.80, and 0.92 (*p* < 0.0001). Concerning the Root Mean Square Error of Approximation (RMSEA), the value was 0.091 (*p* < 0.0001). Figure 2 shows the factor weighting value results for the four Knowledge, Attitude, Nurtures, and Enabling subscales in the standard estimation mode.

The minimum loading factor for varimax rotation in the knowledge subscale was 0.45, the attitude was 0.46, nurtures was 0.45, and enabling was 0.43 (Table 6).

In fitness indicators of P2LQ-PW, the CFI (0.9), RMSEA (0.091), SFI (0.8), NFI (0.92) and χ^2^/dF ratio (3.51) showed a confirmable status (*p* < 0.0001).

### Scoring

The scores for this dimension were calculated as the mean of the ratings for the items. However, no score was computed if there was no response on two or more items. High scores indicate that pregnant women are well understood; lower scores indicate the absence of some or all of these qualities. This questionnaire has four factors. Twenty items measure knowledge with yes, no, and I do not know. To the yes option, a score of 2; to the I do not know option, a score of 1; and to the no option, a score of zero is assigned. The score range is 0–40. Attitude, Nurture, and Enabling are measured with 7, 6, and 6 items, respectively, with a 5-point Likert scale from Strongly, Agree to Strongly Disagree. To the strongly agree option, a score of 5, to the agreement option, a score of 4, to the moderately agree option, a score of 3, to the disagree option, a score of 2, and to the strongly disagree option, a score of 1 is assigned. The score range for Attitude is 7–35, and for Nurture and Enabling is 6–30. In addition, social demography is not an integral part of the tool. The items in the questionnaire are as cut-off points.

### Assessment of reliability of the questionnaire

An intraclass correlation coefficient (ICC) evaluated the test-retest reliability. The criterion was managed to 45 persons 1 month after the first evaluation. The following classification was chosen to construe the compromise levels: 0.0–0.20 as low, 0.21–0.40 as fair, 0.41–0.60 as moderate, 0.61–0.80 as significant, and 0.81–1 as nearly complete and perfect [36]. Also, the internal consistency (IC) of P2LQ-PW is accounted for by calculating Cronbachs’ alpha coefficient. The alpha amount of 0.70 or higher was satisfactory [36]. The standard error of measurement (SEM) was calculated to analyze the absolute reliability of the results. Also, IBM SPSS statistics version 24 was used to perform data cleaning and compute reliability indices. *P* values less than 0.05 were considered significant [35]. Further, IBM SPSS version 24 was used for descriptive analysis.

## Results

After completing the validation process, the final P2LQ-PW included ten questions about the baseline characteristics of the participants and 39 content-specific questions. It is necessary to mention that the included ten questions about the baseline characteristics are not part of the scoring and these are social-demographic parameters.

### Baseline characteristics of the participants

The mean age of women was 32.48 ± 12.40 years, and the average BMI was 26.80 ± 6.25. Table 2 represents the baseline characteristics of the participants.

**Table 2:** Baseline characteristics of pregnant women divided by each stage of the construct validity of the questionnaire

Characteristics	Total (***n*** = 450)	Frequency (%)
**Age (mean ± SD)**	32.48 ± 12.40	
- **Gestational age (week)**		
12–20	160	35.5%
21–28	200	44.5%
29–38	90	20%
**Job**		
Self-employed	137	30.4%
Employee	160	35.6%
Unemployed	153	34%
**Educational level**		
Illiterate	60	13.3%
Elementary	140	31.1%
High school	158	35.2%
Graduate Diploma	92	20.4%
**Income**		
Less than household expenses	236 ()	52.5%
Equal to household expenses	214 ()	47.5%
**Spouse’s education level**		
Illiterate	136	30.2%
Elementary	167	37.2%
High school	64	14.2%
Graduate Diploma	83	18.4%
**Spouse’s job**		
Self-employed	216	48%
Employee	145	32.6%
Unemployed	87	19.4%
**Pre-pregnancy weight**		
Less than 110 pound	236	52.4%
110–176 pound	162	36%
More than 176 pound	52	11.6%
**Kind of pregnancy**		
**Wanted**	338	75.2%
**Unwanted**	112	24.8%
**Length of marriage**		
**L**ess than 5 years	178	39.5%
5–10 years	149	33.2%
More than 10 years	123	27.3%

### Results of the face validity of the P2LQ-PW

All items of P2LQ-PW became clear and understandable. The impact score (IS) of items ranged between 3.6 and 4.8. IS of all items is shown in Table 3.

**Table 3:** The measures of face items of the physical literacy questionnaire (P2LQ-PW)

Items	IS	The source^a^ of the items (Literature/ interviews
**Knowledge**		
** During pregnancy, you should not exercise severely other than pre-pregnancy.**	4	L
** Physical activity is essential to prevent being overweight due to pregnancy.**	4.2	L
** Exercise during pregnancy can lead to reduced oxygen supply to the baby.**	4.8	L
** During pregnancy, you should exercise smoothly and without sloping surfaces.**	4.7	L
** Pregnant women during exercise should avoid excessive curvature and hang up.**	4.8	L
** Exercise during pregnancy makes it easy for normal labor.**	4.2	L
** Exercise in pregnancy reduces the risk of birth in a diabetic neonatal.**	4	L
** Doing basic exercises daily will not hurt the mother and baby.**	4.2	L
** Exercise eliminates the risk of possible complications in pregnancy, e.g., back pain, lumbar pain, constipation, and excessive fatigue.**	4.8	L
** During pregnancy, you should stop doing heavy exercises.**	4.2	L
** Before starting exercise during pregnancy, you should have a light stroke to warm up.**	4.8	L
** A physical activity specialist should take stretching and strength during pregnancy.**	4.2	L
** Exercising in pregnancy prevents blood pressure dangers.**	4	L
** Exercise during pregnancy can lead to a return faster maternal postpartum.**	4.2	L
** I try to avoid lifting any weight during pregnancy.**	4.7	L
** Exercising during pregnancy leads to fitness and weight control.**	4.7	L
** For pre-exercise preparation, 15 minutes of gentle relaxation movements are required.**	4.8	L
** Before conducting any exercise at this time, consultation with the doctor is required.**	4.7	L
** Exercise reduces the risk of musculoskeletal discomfort.**	4.7	L
** In the third period of pregnancy, exercise intensity should be reduced.**	4.2	L
**Attitude**		
** I believe that I can efficiently deal with problems such as gestational diabetes with exercise.**	4.8	L
** I believe that exercising during pregnancy reduces my fatigue due to my pregnancy.**	4.2	L
** I believe that I can work out stress and anxiety from childbirth during this period.**	4	L
** I believe exercising during pregnancy helps with my daily activities.**	4.2	L
** I believe that I can easily maintain my fitness by exercising during pregnancy.**	4.8	L
** I believe that by exercising, I can reduce postpartum depression.**	4.2	L
** I believe that if I exercise during pregnancy, I can have a more comfortable delivery.**	4	L
**Nurtures**		
** If my husband attends exercise classes during pregnancy with me, It is easier to convince him that exercise is not dangerous.**	4.7	L
** If my own family and husband’s family attend exercise classes during pregnancy, I can persuade them that exercise is not dangerous.**	4.2	L
** I encourage friends and acquaintances to exercise during pregnancy**	4	L
** If there is a group discussion to exchange information about pregnant women after a pregnancy class, I tend to attend a pregnancy class.**	4	L
** If my family is in physical education classes …**	4.8	L
** If a skilled and informed person answers midwifery questions after a pregnancy class, I would be more eager to participate in pregnancy classes.**	4.7	L
** If my family would advise me to do exercise …**	3.9	L
** If my doctor advises me to exercise during pregnancy, I will do that.**	4	L
**Enabling**		
** If there is an excellent place to exercise, it is easier to exercise during pregnancy.**	4.8	L
** If classes are held free, I can use these classes more easily.**	4.2	L
** If there is a physical activity area near my residence, I can get better and more comfortable with pregnancy classes.**	3.9	L
** If I have a training CD in the centers, I can do more exercise at home.**	4.2	L
** If there are enough facilities, such as a buffet, a washbasin, etc., in pregnancy classes, I always try to take part in pregnancy classes**	4.8	L
** If exercise classes occur at different times throughout the day, I can more easily participate in physical activity classes.**	4	L

^a^literature(L) or interview(I)

### Results of the content validity of the P2LQ-PW

Table 4 demonstrates the items’ CVI and CVR, indicating their ranges as 0.80–1.00 and 0.52–1.00, respectively.

**Table 4:** The measures of content validity of the items of the physical literacy questionnaire (P2LQ-PW)

Items	CVR (%)	CVI (%)
**Knowledge**		
** During pregnancy, you should not exercise severely other than pre-pregnancy.**	84	100
** Physical activity is essential to prevent being overweight due to pregnancy.**	78	92
** Exercise during pregnancy can lead to reduced oxygen supply to the baby.**	100	100
** During pregnancy, you should exercise smoothly and without sloping surfaces.**	84	88
** Pregnant women during exercise should avoid excessive curvature and hang up.**	78	82
** Exercise during pregnancy makes it easy for normal labor.**	84	100
** Exercise in pregnancy reduces the risk of birth in a diabetic neonatal.**	82	88
** Doing basic exercises daily will not hurt the mother and baby.**	82	88
** Exercise eliminates the risk of possible complications in pregnancy, e.g., back pain, lumbar pain, constipation, and excessive fatigue.**	78	88
** During pregnancy, you should stop doing heavy exercises.**	82	92
** Before starting exercise during pregnancy, you should have a light stroke to warm up.**	88	100
** A physical activity specialist should take stretching and strength during pregnancy.**	92	86
** Exercising in pregnancy prevents blood pressure dangers.**	84	77
** Exercise during pregnancy can lead to a return faster maternal postpartum.**	82	86
** I try to avoid lifting any weight during pregnancy.**	88	92
** Exercising during pregnancy leads to fitness and weight control.**	89	96
** For pre-exercise preparation, 15 minutes of gentle relaxation movements are required.**	87	92
** Before conducting any exercise at this time, consultation with the doctor is required.**	92	88
** Exercise reduces the risk of musculoskeletal discomfort.**	90	100
** In the third period of pregnancy, exercise intensity should be reduced.**	85	88
**Attitude**		
** I believe that I can efficiently deal with problems such as gestational diabetes with exercise.**	100	100
** I believe that exercising during pregnancy reduces my fatigue due to my pregnancy.**	88	92
** I believe that I can work out stress and anxiety from childbirth during this period.**	74	88
** I believe exercising during pregnancy helps with my daily activities.**	84	95
** I believe that I can easily maintain my fitness by exercising during pregnancy.**	84	98
** I believe that by exercising, I can reduce postpartum depression.**	84	92
** I believe that if I exercise during pregnancy, I can have a more comfortable delivery.**	85	98
**Nurtures**		
** If my husband attends exercise classes during pregnancy with me, It is easier to convince him that exercise is not dangerous during pregnancy.**	85	100
** If my own family and my husband’s family attend exercise classes during pregnancy, I can persuade them that exercise is not dangerous.**	88	92
** I encourage friends and acquaintances to exercise during pregnancy**	82	8
** If there is a group discussion to exchange information about pregnant women after a pregnancy class, I tend to attend a pregnancy class.**	88	92
** If my family is in physical education classes …**	52^a^	67^a^
** If a skilled and informed person answers midwifery questions after a pregnancy class, I would be more eager to participate in pregnancy classes.**	90	100
** If my family would advise me to do exercise …**	32^a^	64^a^
** If my doctor advises me to exercise during pregnancy, I will do that.**	92	88
**Enabling**		
** If there is an excellent place to exercise, it is easier to exercise during pregnancy.**	88	92
** If classes are held free, I can use these classes more easily.**	78	82
** If there is a physical activity area near my residence, I can get better and more comfortable with pregnancy classes.**	92	100
** If I have a training CD in the centers, I can do more exercise at home.**	95	88
** If there are enough facilities, such as a buffet, a washbasin, etc., in pregnancy classes, I always try to take part in pregnancy classes**	86	96
** If exercise classes occur at different times throughout the day, I can more easily participate in physical activity classes.**	86	92

^a^These items were omitted

### Results of the construct validity of the P2LQ-PW

Among 450 completed P2LQ-PW, 12 incomplete ones were excluded, and EFA and CFA were implemented based on the 212 and 220 completed ones.

### Results of the exploratory factor analysis

The generalized EFA was performed due to the binary measurement level of the items. EFA is used to determine correlations among a set of observed binary ones. This study extracted the factors using oblique GEOMIN rotation procedures and the maximum likelihood estimation method. Additionally, binary-type items were loaded in four sub-scales of Awareness, Attitude, Nurture, and Enabling physical activity in pregnant women, with 20, 7, 6, and 6 items. The variations in the preventive behavior of physical activity were predicted by changing their awareness, attitude, nurture, and enabling scores. The predictive power of awareness, attitude, nurture, and enabling scales was 38.43, 25.89, 14.73, and 15.81%. Table 5 summarizes the loadings of the extracted factors.

**Table 5:** The results of the measurement model divided by each construct of the physical literacy questionnaire (P2LQ-PW)

Scale	χ2	χ2/df	*P*	CFI	TLI	RMSEA (95% CI)	Eigenvalue	%variance	Cumulative % variance
Awareness	240.18	2.00	< 0.001	.940	.932	.079 (.057; .081)	9.41	15.89	15.89
Attitude	46.26	1.21	0.183	.990	.987	.038 (.001; .061)	4.93	14.81	30.70
Nurtures	221.60	3.06	< 0.001	.976	.953	.72 (.064; .093)	5.65	12.73	43.43
Enabling	42.45	1.64	0.019	.991	.988	.062 (.023; .083)	5.26	15.81	59.24

χ2, chi-square; χ2/df, normed chi-square, *CFI* Comparative Fit Index, *TLI* Tucker, and Levis Index, *RMSEA* Root mean square error of approximation

The results of KMO, Bartlett′s tests, and MSA for factors of the P2LQ-PW are shown in Table 6.

**Table 6:** The results of exploratory factor analysis of KMO, Bartlett′s tests, MSA of the physical literacy questionnaire (P2LQ-PW)

Scale	KMO	BT	MSA
Awareness	0.949	3016.12	0.945
Attitude	0.939	2994.88	0.965
Nurtures	0.910	1816.92	0.941
Enabling	0.884	1518.71	0.924

*KMO* Kaiser-Meyer-Olkin, *BT* Bartlett′s test, CFI, *MSA* Measures of Sampling Adequacy

### Results of the confirmatory factor analysis

Confirmatory factor analysis was done for a 10-degree Likert scale and 29 binary items. In this process, 39 remaining items were considered indicators, and four latent variables were considered constructs. All the relationships between the constructs (awareness, attitude, nurtures, and enabling) and items (or 39 indicators) were significant (All *p* < 0.05). The results indicated a good fitness of the model. The model and the loadings of the extracted factors are presented in Fig. 2.

Additionally, the results of CFA, including T-statistics and factor loadings, were reported in Table 7.

**Table 7:** The results of the final confirmatory factor analysis of the physical literacy questionnaire (P2LQ-PW) (model factor loadings)

Items	F1	F2	F3	F4
**During pregnancy, you should not exercise severely other than pre-pregnancy.**	0.95	–	–	–
**Physical activity is essential to prevent being overweight due to pregnancy.**	0.79	–	–	–
**Exercise during pregnancy can lead to reduced oxygen supply to the baby.**	0.45	–	–	–
**During pregnancy, you should exercise smoothly and without sloping surfaces.**	0.48	–	–	–
**Pregnant women during exercise should avoid excessive curvature and hang up.**	0.94	–	–	–
**Exercise during pregnancy makes it easy for normal labor.**	0.94	–	–	–
**Exercise in pregnancy reduces the risk of birth in a diabetic neonatal.**	0.78	–	–	–
**Doing basic exercises daily will not hurt the mother and baby.**	0.45	–	–	–
**Exercise eliminates the risk of possible complications in pregnancy, e.g., back pain, lumbar pain, constipation, and excessive fatigue.**	0.55	–	–	–
**During pregnancy, you should stop doing heavy exercises.**	0.84	–	–	–
**Before starting exercise during pregnancy, you should have a light stroke to warm up.**	0.74	–	–	–
**A physical activity specialist should take stretching and strength during pregnancy.**	0.45	–	–	–
**Exercising in pregnancy prevents blood pressure dangers.**	0.93	–	–	–
**Exercise during pregnancy can lead to a return faster maternal postpartum.**	0.92	–	–	–
**I try to avoid lifting any weight during pregnancy.**	0.65	–	–	–
**Exercising during pregnancy leads to fitness and weight control.**	0.97	–	–	–
**For pre-exercise preparation, 15 minutes of gentle relaxation movements are required.**	0.85	–	–	–
**Before conducting any exercise at this time, consultation with the doctor is required.**	0.43	–	–	–
**Exercise reduces the risk of musculoskeletal discomfort.**	0.90	–	–	–
**In the third period of pregnancy, exercise intensity should be reduced.**	0.93	–	–	–
**I believe that I can efficiently deal with problems such as gestational diabetes with exercise.**	–	0.85	–	–
**I believe that exercising during pregnancy reduces my fatigue due to my pregnancy.**	–	0.89	–	–
**I believe that I can work out stress and anxiety from childbirth during this period.**	–	0.94	–	–
**I believe exercising during pregnancy helps with my daily activities.**	–	0.91	–	–
**I believe that I can easily maintain my fitness by exercising during pregnancy.**	–	0.46	–	–
**I believe that by exercising, I can reduce postpartum depression.**	–	0.93	–	–
**I believe that if I exercise during pregnancy, I can have a more comfortable delivery.**	–	0.48	–	–
**If my husband attends exercise classes during pregnancy with me, It is easier to convince him that exercise is not dangerous during pregnancy.**	–	–	0.52	–
**If my own family and my husband’s family attend exercise classes during pregnancy, I can persuade them that exercise is not dangerous.**	–	–	0.78	–
**I encourage friends and acquaintances to exercise during pregnancy.**	–	–	0.83	–
**If there is a group discussion to exchange information about pregnant women after a pregnancy class, I tend to attend a pregnancy class.**	–	–	0.90	–
**If a skilled and informed person answers midwifery questions after a pregnancy class, I would be more eager to participate in pregnancy classes.**	–	–	0.45	–
**If my doctor advises me to exercise during pregnancy, I will do that.**	–	–	0.67	–
**If there is an excellent place to exercise, it is easier to exercise during pregnancy.**	–	–	–	0.86
**If classes are held free, I can use these classes more easily.**	–	–	–	0.86
**If there is a physical activity area near my residence, I can get better and more comfortable with pregnancy classes.**	–	–	–	0.88
**If I have a training CD in the centers, I can do more exercise at home.**	–	–	–	0.48
**If there are enough facilities, such as a buffet, a washbasin, etc., in pregnancy classes, I always try to participate in pregnancy classes.**	–	–	–	0.60
**If exercise classes occur at different times throughout the day, I can more easily participate in physical activity classes.**	–	–	–	0.43

F1: Knowledge, F2: Attitude, F3: Nurtures, F4: Enabling

Based on the findings from the participation of 450 pregnant women, the descriptive statistics of the subscales (Knowledge, Attitude, Nurture, and Enabling) analysis of the psychometric properties of the Theory-Based Perceptions Concerning the Physical Literacy Questionnaire for Pregnant Women is shown in Table 8.

**Table 8:** Descriptive statistics of the Subscales analysis of the psychometric study of the physical literacy questionnaire (PA2L-PW) (*n* = 450 pregnant women)

Subscales	No of items	Mean ± SD	Skewness	Cronbach’s alpha	ICC	CR
**Knowledge**	20	86.8 **±** 12.8	−1.71	0.96	0.76	0.98
**Attitude**	7	78.5 **±** 14.7	−0.81	0.97	0.79	0.94
**Nurture**	6	75.4 **±** 16.5	−0.70	0.94	0.83	0.89
**Enabling**	6	58.1 **±** 25.7	− 0.40	0.89	0.89	0.90

### Results of the reliability of the questionnaire

The reliability measures of the research P2LQ-PW are presented in Table 8.

## Discussion

The P2LQ-PW is considered the first theory-based questionnaire focused on pregnant women, which was designed and validated for evaluating pregnant women’s perceptions concerning physical literacy and assessing psychometric properties and attributes. According to the results, it is suggested that research design all the constructs of the PEN-3 cultural model that consist of perception, enabling, and nurture factors. In this research, various assumptions were created due to direct and indirect views about the fundamental cognitive structures, and neither view is complete. When several procedures are knapping a similar construct, degrees are anticipated to be affirmatively associated, so it is suggested that both be contained in a questionnaire with PEN-based [37].

For the perceptions (knowledge, attitude), nurtures, and enabling factors, the development sample’s internal consistency of reliabilities was above 0.80. For the perceptions (knowledge, attitude), nurtures, and enabling factors, the test-retest correlation coefficients were about 0.90 in the intended sample. These rates were checked sufficiently [38].

This survey used an approach that is more acceptable for pregnant women. In addition, the reliabilities were higher than those received in primary function with first pregnant women that contained perceptional factors (attitude, knowledge) [36]. However, the internal consistency of attitude about the aim of being exercised (0.63) was sufficient but not broad. However, it is higher than the test-retest reliability presented in previous work for pregnant women [36]. It is eminence that the reliability of the test-retest in this study was managed 1 month after the first data collection. In this study, the ICC for full-scale physical literacy was 0.92, higher than the questionnaire in previous research among pregnant women [36].

This study is considered to design and validate a questionnaire (P2LQ-PW), which can be used for variables based on the theoretical framework [39]. Moreover, various indicators, such as χ^2^/df ratio, CFI, GFI, and RMSEA, verified the compatibility of the models. The results regarding the (NFI, RMSEA, and CFI) were higher than expected. This finding also demonstrated that each of the four sub-constructs in the questionnaire (P2LQ-PW) fit appropriately within the PEN-3 cultural model framework.

Empirical results from the Cronbach’s Alpha, test-retest, and interrater reliability confirmed that, within the four subconstructs, the P2LQ-PW shows acceptable internal consistency (ranging from 0.92 to 0.97); provides reliable results over repeated administrations (ranging from 0.97 to 0.99) at the pregnant women during pregnancy. The reliability of the questionnaires was evaluated in previous studies from test-retest stability and internal consistency. For example, Satter et al. evaluated different instrument based on previous literature. Their indicated reliability ranged from 0.70 to 0.80 [38]. The recent physical literacy questionnaire demonstrated a good correlation with the accelerometer that was used for physical activity assessments for whole times for being active (*r* ≥ 0.50) but not for all of the physical activity energy expenditure (PAEE) and other approximations of physical activity [36]. Further, the reliability of the results was confirmed by the ICC (at least 0.885), CR (0.895), and SEM (5.448) of the constructs, which demonstrates the acceptable internal consistency and stability of the results related to P2LQ-PW [33, 40].

Chasan-Taber et al. designed and validated the reliability of the physical activity questionnaire (PPAQ) within 54 pregnant women [41].

Therefore, according to the authors, the P2LQ-PW is suitable for various potential applications to measure physical literacy and its main determinants among Persian pregnant women. Besides, the P2LQ-PW tool is not suitable for non-pregnant women. One unique feature of P2LQ-PW is the reliability and validity of its subscales, which include knowledge and attitude, nurtures, enabling, and physical activity behavior. These sub-constructs may be measured, evaluated, and modified by potential change strategies, promoting physical activity during pregnancy, and health promotion.

Some limitations of this study must be noted. First, data were only collected from the second and third trimester pregnant women attending health centers in capital Tehran’s region 5; and other independent health centers, other gestational ages during pregnancy, did not enroll to study the generalizability of results to all of the society may be confined. Subscales of the questionnaire (P2LQ-PW) were limited to the determinants of behavior in the PEN-3 cultural model and the other constructs (psychological determinants of behavior) to decrease the questions’ burden on participants not using. Notwithstanding the limitations explained, the P2LQ-PW is the instrument of validity and reliability to assess women’s exercise behavior through pregnancy in Persia. Future studies that apply this questionnaire could help overcome this kind of problem.

## Conclusion

P2LQ-PW is the first standard tool based on the PEN-3 cultural model that researchers can utilize to gather data and conduct the desired education interventions to change physical activity behavior in pregnant women. Co-operating the public and governmental institutions is required to increase physical literacy in pregnant women worldwide, especially in the Middle East and in different transmission ways. The P2LQ-PW was consistent and reliable within its knowledge, attitude, nurturing, and enabling subscales. However, future attempts are carried out to assess whether P2LQ-PW applies in the first trimester of pregnancy in pregnant women attending various community-based settings, including the health services center.

### Strengths of the study

P2LQ-PW is the first theory-driven questionnaire in physical literacy and focuses on real problems, barriers, and facilitators of preventive behavior in pregnant women. In this study, the triangulation method generated the questionnaire’s items. The items were created by the researcher’s field experiences, literature review, and interviews with various stakeholders (experts, health educationists, and midwives).

### Limitations of the study

In this study, 12% of the participants were uneducated. The researcher had to explain all questions to the pregnant women and complete the research questionnaires by himself.

Each question was read in the language of the pregnant women, and the answers were checked with the pregnant women themselves. Therefore, this required more time for an explanation for each participant. Moreover, this study included pregnant women aged 18 to 38 years. However, Smaller participants may have very different experiences and perspectives and may face many obstacles and different perceptions. Unfortunately, we do not have access to this group from the health center. The high number of participants with low education reduces the validity of the questionnaire for the general population, and this issue can be considered a study limitation.

### Implications

The P2LQ-PW, designed and validated, can be employed by all health educationists to develop educational interventions that are related to perceptive, nurturing, and enabling factors in the cultural context.

## Supplementary Information

**Additional file 1.**

## Data Availability

All data generated during this research are included in this article.

## Abbreviations

LMICs: Low- and middle-income countries
SES: Socioeconomic status
GDM: Gestational diabetes mellitus
ACOG: American college of obstetrics and gynecology
QOL: quality of life
WHO: World Health Organization
CFI: Comparative Fit Index
NFI: Normed Fit Index
GFI: Goodness of fit index
RMSEA: Root Mean Square Error of Approximation
