# Supplementary material for: Design and validation of theory-based perceptions concerning the physical literacy questionnaire for pregnant women (P2LQ-PW)
Source: BMC Public Health. 2022 Oct 23;22:1955. doi: 10.1186/s12889-022-14204-7 (PMC9588218; doi:10.1186/s12889-022-14204-7)
Supplement: Supplementary file 1 — Additional file 1. [file 12889_2022_14204_MOESM1_ESM.docx]

Completion date of the questionnaire: ID number of your workplace:

| The following questionnaire has been designed to investigate the Development and Psychometrics of a Theory-Based Physical Activity Assessment Questionnaire for the Pregnant Women (PA2Q-PW). Your cooperation will help us to take useful steps in improving physical activity-related behaviors. Surely, the information will be kept completely confidential. They will be used for only research work. Appreciate your invaluable time and cooperation in completing this questionnaire. |
| --- |

**Age (year):**

**Gestational age:**

**Job:**

**Self-employed  employee  Unemployed **

**Education level:**

**Illiterate  Elementary  High school Graduate diploma **

**Income:**

**Less than household expenses  Equal to household expenses**

**Spouse's education level:**

**Illiterate  Elementary  High school Graduate diploma **

**Spouse's job:**

**Self-employed  employee  Unemployed **

**Pre-pregnancy weight:**

**Less than 110 pounds  110-176 pounds more than 176 pounds**

**Kind of Pregnancy**

**Wanted Unwanted**

**Length of marriage No**

**5-10 years less than 5 years more than 10 years**

**Responding to questions from 1 to 20, please select the correct choice (Yes / No/ I do not know)**

| **Item** | **YES** | **NO** | **I do not know** |
| --- | --- | --- | --- |
| During pregnancy, you should not exercise severely other than pre-pregnancy. |  |  |  |
| Physical activity is essential to prevent overweight due to pregnancy. |  |  |  |
| Exercise during pregnancy can lead to reduced oxygen supply to the baby. |  |  |  |
| During pregnancy, you should exercise smoothly and without sloping surfaces. |  |  |  |
| Pregnant women during exercise should avoid excessive curvature and hang up. |  |  |  |
| Exercise during pregnancy makes it easy for normal labor. |  |  |  |
| Exercise in pregnancy reduces the risk of birth in a diabetic neonatal. |  |  |  |
| Doing basic exercises daily will not hurt the mother and baby. |  |  |  |
| Exercise eliminates the risk of possible complications in pregnancy, e.g., back pain, lumbar pain, constipation, and excessive fatigue. |  |  |  |
| During pregnancy, you should stop doing heavy exercises. |  |  |  |
| Before starting exercise during pregnancy, you should have a light stroke to warm up. |  |  |  |
| A physical activity specialist should take stretching and strength during pregnancy. |  |  |  |
| Exercising in pregnancy prevents blood pressure dangers. |  |  |  |
| Exercise during pregnancy can lead to return faster maternal postpartum. |  |  |  |
| I try to avoid lifting any weight during pregnancy. |  |  |  |
| Exercising during pregnancy leads to fitness and weight control. |  |  |  |
| For pre-exercise preparation, 15 minutes of gentle relaxation movements are required. |  |  |  |
| Before conducting any exercise at this time, consultation with the doctor is required. |  |  |  |
| Exercise reduces the risk of musculoskeletal discomfort. |  |  |  |
| In the third period of pregnancy, the intensity of exercise should be reduced. |  |  |  |

**Responding to questions 21 to 39, please choose one of the options (Strongly Agree, Agree, Moderately Agree, Disagree, or strongly disagree)**

| **Item** | **Strongly Agree** | **Agree** | **Moderately agree** | **Disagree** | **Strongly disagree** |
| --- | --- | --- | --- | --- | --- |
| I believe that with exercise, I can efficiently deal with problems such as gestational diabetes. |  |  |  |  |  |
| I believe that exercising during pregnancy reduces my fatigue due to my pregnancy. |  |  |  |  |  |
| I believe that I can work out stress and anxiety from childbirth during this period. |  |  |  |  |  |
| I believe exercising during pregnancy helps with my daily activities. |  |  |  |  |  |
| I believe that I can easily maintain my fitness by exercising during pregnancy. |  |  |  |  |  |
| I believe that by exercising, I can reduce postpartum depression. |  |  |  |  |  |
| I believe that if I exercise in pregnancy, I can have a more comfortable delivery. |  |  |  |  |  |
| If my husband attends exercise classes during pregnancy with me, It is easier to convince him that exercise is not dangerous during pregnancy. |  |  |  |  |  |
| If my own family and my husband's family attend exercise classes during pregnancy, I can persuade them that exercise is not dangerous. |  |  |  |  |  |
| I encourage friends and acquaintances to exercise during pregnancy |  |  |  |  |  |
| If there is a group discussion to exchange information about pregnant women after a pregnancy class, I have a greater tendency to attend a pregnancy class. |  |  |  |  |  |
| If a skilled and informed person answers midwifery questions after a pregnancy class, I would be more eager to participate in pregnancy classes. |  |  |  |  |  |
| If my doctor advises me to do exercise in pregnancy, I will do that. |  |  |  |  |  |
| If there is an excellent place to exercise, it is easier to exercise in pregnancy. |  |  |  |  |  |
| If classes are held free, I can use these classes more easily. |  |  |  |  |  |
| If there is a physical activity area near my residence, I can get better and more comfortable with pregnancy classes. |  |  |  |  |  |
| If I have a training CD in the centers, I can do more exercise at home. |  |  |  |  |  |
| If there are enough facilities, such as a buffet, a washbasin, etc., in pregnancy classes, I always try to take part in pregnancy classes |  |  |  |  |  |
| If exercise classes occur at different times throughout the day, I can more easily participate in physical activity classes. |  |  |  |  |  |
